# Vortex-Induced Vibrations of an Elastic Micro-Beam with Gas Modeled by DSMC

**DOI:** 10.3390/s23041933

**Published:** 2023-02-09

**Authors:** Kiril Shterev, Emil Manoach, Simona Doneva

**Affiliations:** 1 Institute of Mechanics, Bulgarian Academy of Sciences, Sofia 1113, Bulgaria; 2 Institute of Information and Communication Technologies, Bulgarian Academy of Sciences, Sofia 1113, Bulgaria

**Keywords:** vortex-induced vibrations, elastic micro-beam, DSMC, resonance curve

## Abstract

The fluid–structure interaction is one of the most important coupled problems in mechanics. The topic is crucial for many high-technology areas. This work considers the interaction between an elastic obstacle and rarefied gas flow, seeking specific problems that arise during this interaction. The Direct Simulation Monte Carlo method was used to model the rarefied gas flow and the linear Euler–Bernoulli beam theory was used to describe the motion of the elastic obstacle. It turned out that the vibrations caused by the gas flow could provoke a resonance-like phenomenon when the frequency of vortex shedding of the flow was close to the natural frequency of the beam. This phenomenon could be useful in certain high-technology applications.

## 1. Introduction

The fluid–structure interaction (FSI) is one of the most important and complicated coupled problems in structural dynamics. It is a challenge not only for its difficulty but also for its importance. Basic aspects of this field are deeply considered in the literature [1,2]. Historically, the first works on FSI were devoted to pipes conveying fluid, large offshore structures, structures in energy, and chemical technology.

Recently, the interaction of micro-sized flows with elastic elements has become an important part of many technological devices. These include micro-electromechanical systems (MEMS) diaphragm drop ejectors, atomic force microscopy (AFM), air-film damping devices, sensors for monitoring harmful environmental gases or breath gas analysis, devices for detecting flow separation into a boundary layer, and small energy harvesting devices using vortex-induced vibration (VIV). Authors have used different approaches and simplifications to calculate these problems.

An attempt was made to solve the problem of strong coupling of the equations describing the motion of an elastic plate and fluid flow in [3]. The authors used the finite difference method and developed a computer code for a plate vibration that they connected with the commercial 3D Flow program. Following that, they studied the dynamic behavior of the MEMS diaphragm drop ejector. In [4], the authors used the finite element method, and coupled the developed computer code with the commercial computational fluid dynamics (CFD) solver FLUENT. During the solution, the interface motion was transferred through the use of the moving mesh capability of the CFD solver.

Problems involving the interaction of gas flows with obstacles were considered in [5,6,7]. In [5], a novel micro-gas flow sensor comprising four silicon nitride/silicon wafer cantilever beams was considered. The velocity of the air flow was determined by measuring the change in resistance of the piezoresistors patterned on the surface of each cantilever beam. The authors fabricated such a sensor and tested it. The theoretical model is extremely simple and does not consider any coupling between the fields. A simple model of FSI was also used in [6]. The micro-beam considered in this work is presented as a simple harmonic oscillator and the influence of the gas is considered as an added mass. This work is a good example of using a micro-beam in an AFM to measure gas properties. The behavior of the cantilever in AFM was also considered in [7]. The effect of fluid loading was analyzed by a systems/feedback approach. The linear Euler–Bernoulli beam theory was used to describe the cantilever’s behavior. Its response is presented as a series of products of the natural modes of the beam and time-dependent functions. The effect of the fluid on the structure is twofold: fluctuating forces result in random motion, and hydrodynamic forces (viscous and inertial) directly affect the dynamics of the cantilever. The thermal response was analyzed as well, and regimes at low Reynold numbers were considered.

On the other hand, air-film damping plays a significant role in the dynamic response of many micro-fabricated devices with a movable mass suspended by various bearing mechanisms. The problem has been considered by many authors [8,9,10,11,12]. Most studies have considered slow fluid velocity and used a continuum model based on Navier–Stokes equations. Some authors have tried to present an easy-to-use analytical model [8], while others have used numerical methods to seek more accurate solutions. Seeking a more accurate numerical solution, Wang et al. [9] and Wang et al. [10] used finite element-based multiphysics simulation and the ALE DUGKS solution procedure, respectively. In the ALE-type DUGKS, the velocity of the mesh motion is introduced in the Boltzmann–BGK equation to modify the net flux of a cell interface (see [13]). Diab and Lakkis [11,12] used Direct Simulation Monte Carlo (DSMC, a molecular approach) to model rarefied gas for micro-detection of different kinds of gas film, but the micro-beam motion was presupposed with its vibration frequency and velocity. The equation of beam motion was not considered. Even the main beam’s characteristics, such as thickness, length, and material properties, were not used in the theoretical analysis. In this sense, the problem of full coupling of the two fields (fluid and structural vibration) was not considered and the beam was not directly influenced by the gas flow.

Another trend is using sensors based on micro- and nanostructures. Such sensors have been developed to monitor harmful gases in the environment or breath gas analysis as part of health diagnostics [14,15]. Oosthuizena, Motaung, and Swart [14] evaluated CO gas sensing at room temperature (20 °C) in the presence of dry air and various relative humidity levels, using undoped p-type CuO nanoplatelets synthesized by hydrothermal reactions, without any surfactants. Kim et al. [15] presented a 2 × 4 sensor array with a micro-heater and ceramic chip. The sensor array detected CH_3_COCH_3_, NO_2_, and H_2_S as biomarkers for breath gas analysis.

Flow separation is another important part of fluid flow characteristics because it changes a device’s flow regime and behavior. For airplane flow, separation leads to a sudden drop-off in lift force that could be very dangerous. Different sensors have been developed to study and monitor flow separation [16,17]. Corke et at. [16] presented a boundary layer separation detection approach that used a plasma actuator. Sturm, Dumstorff, and Busche [17] studied sensors based on changes in temperature distribution on a membrane (calorimetric principle). The study presented here can be used to develop a sensor for real-time monitoring of gas properties in the boundary layer of operating devices and vehicles.

In recent years, attention has turned to the energy harvesting possibility of small structures. A possible source of energy is VIV. VIV refers to motion of the structure induced by irregularities in the interacting external flow. Classical investigations on VIV were related to preventing damage to structures [18,19], but VIV can be used as a source of energy for different types of wireless sensor networks for the Internet of Things. Construction of such a device involves a small cylinder with a diameter from a couple of millimeters to a couple of centimeters attached to a MEMS with an appropriate piezoelectric element [20,21]. The numerical part of the investigation uses CFD simulations based on continuum models. Continuum models of fluids, such as the Navier–Stokes equations, have decreased accuracy when the non-equilibrium effects for a considered problem are significant, i.e., when the size of the objects approaches the local mean free path of the molecules. In such cases, appropriate models have to be used.

In this paper, we used the advantage of mathematical modeling to investigate new phenomena. The results can provide a basis for developing new micro-devices. Theoretical studies seeking new possibilities and answers to important questions are common in microfluidics, where flows are in slip-flow and transition regimes. A slip-flow regime is within the range 0.001 ≤ Kn ≤ 0.1 and a transition regime is in the range 0.1 ≤ Kn ≤ 10 [22], where Kn is the Knudsen number, defined as a ratio of the mean free path (*λ*_∞_) to the macroscopic reference length (*L*), Kn = *λ*_∞_/*L*. Navier–Stokes equations can be used with slip boundary conditions in the slip-flow regime, while their deviation is significant in the transition regime. Despite these common assumptions, it was proved that the correctness of Navier–Stokes equations depends on the particular problem and its specifics. Mohammadzadeh et al. [23] proved that in a cavity flow discrepancy between Navier–Stokes and DSMC, the results are larger near the tip corners of the cavity, where non-equilibrium effects are dominant even for Kn equal to 0.005, which is close to the continuum regime.

The FSI of microdevices includes non-equilibrium gas effects on surfaces that have to be taken into account, especially for continuously vibrating elastic beams. On the other hand, DSMC is widely used as a benchmark to determine the correctness of other approaches in slip-flow and transition regimes [24], and the hydrodynamic effects discovered by DSMC were confirmed later [25,26]. These motivated us to develop a fully coupled approach where DSMC simulates micro-gas flow, which considers direct particle interactions with the surface of the elastic element [27]. We used this hybrid approach in the present study. The sizes of micro-devices are from 1 µm to 1 mm, and the area of interest for the processes can be much smaller, which increases the local Knudsen number and requires an appropriate approach. The measurements of and experiments with such small and fast processes could be difficult. The transition times of fluid flows are less than 10^−6^ to 10^−5^ s, and the important changes in a flow happen within less than 5 × 10^−7^ s. Such processes were studied in this paper. The results show possible advantages of developing micro-devices using elastic micro-beams.

**Figure 1:**
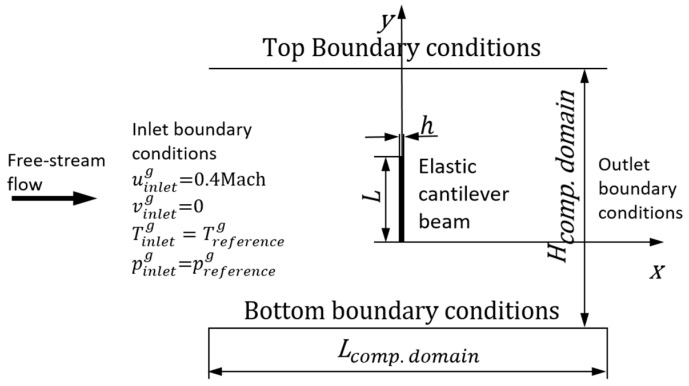
Geometry of flow past elastic cantilever beam placed normal to free stream.

In this work, within the framework of FSI, we studied the interaction between VIV and the first natural frequency of the elastic micro-beam. We considered rarefied gas past an elastic beam normal to the free stream (Figure 1). Behind the beam, a Karman vortex street was formed that induces vibrations in the beam. DSMC was used to model the gas, and the elastic beam was modelled by linear Euler–Bernoulli beam theory. We used the hybrid numerical approach described in detail in [27]. To our knowledge, this is the first investigation of this type of a fully coupled FSI using a molecular approach (DSMC) to simulate the gas.

## 2. Mathematical Models and Validation

The DSMC method was used to model rarefied gas flow. DSMC is a widely used approach in rarefied gas dynamics that was initially developed by Bird [28] to simulate supersonic and hypersonic flows, but has found excellent application in microfluidics in recent decades. As discussed in the Introduction, DSMC has notable advantages in modelling non-equilibrium effects on the surface in a slip regime and is widely used as a benchmark to determine the validity of other models. This motivated us to use DSMC in our study. The linear Euler–Bernoulli beam theory was applied to describe the beam motion. This hybrid numerical approach is described in detail in [27]. The variables related to the gas were denoted with superscript g.

### 2.1. DSMC

Flow past a flat plate placed normal to the free stream as a validation test case was analyzed. This corresponds to a standard problem considered in continuum fluid mechanics, but here the gas was modeled by a molecular approach (DSMC) accounting for the specifics of rarefied flows, especially non-equilibrium effects on the surface. The selected test case used a rigid flat plate, while the VIV study described in the next section (Section 3) used an elastic beam. The displacements and velocities of the elastic beam are small and their influence on the macro characteristics of the rarefied gas flow is negligible, and small changes of thickness (*h*) have a negligible influence on the flow. That makes the cases of rigid cantilever (flat plate) and elastic cantilever beam very close from the point of view of the fluid flow.

**Figure 2:**
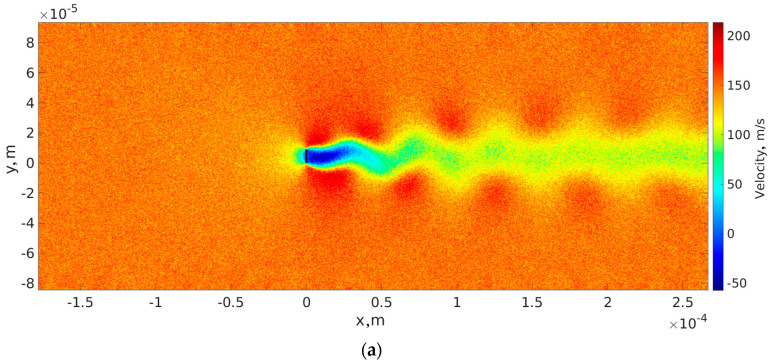

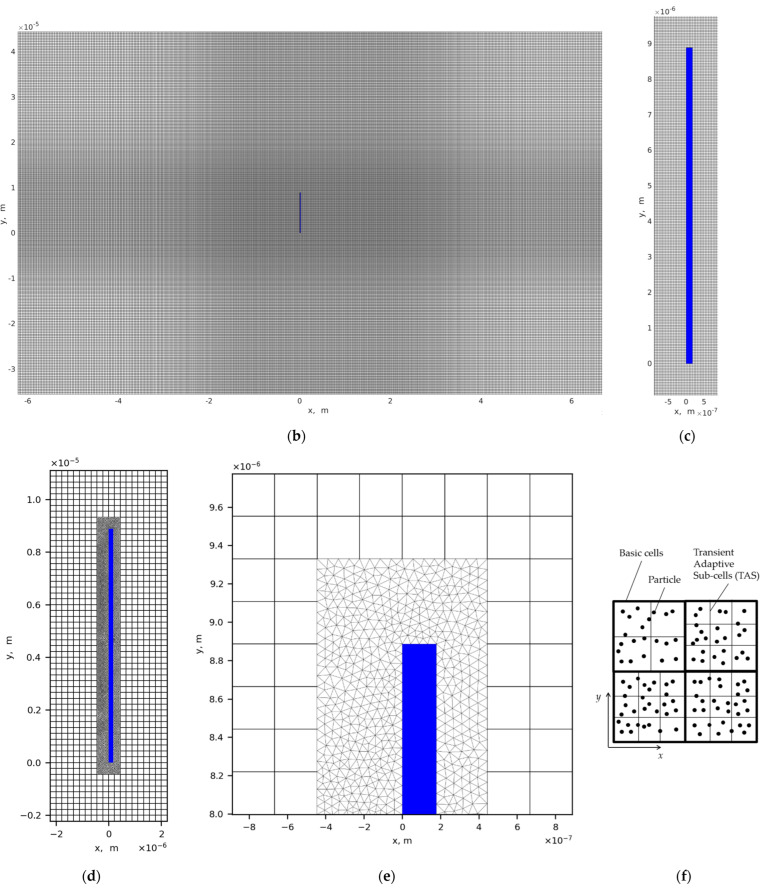

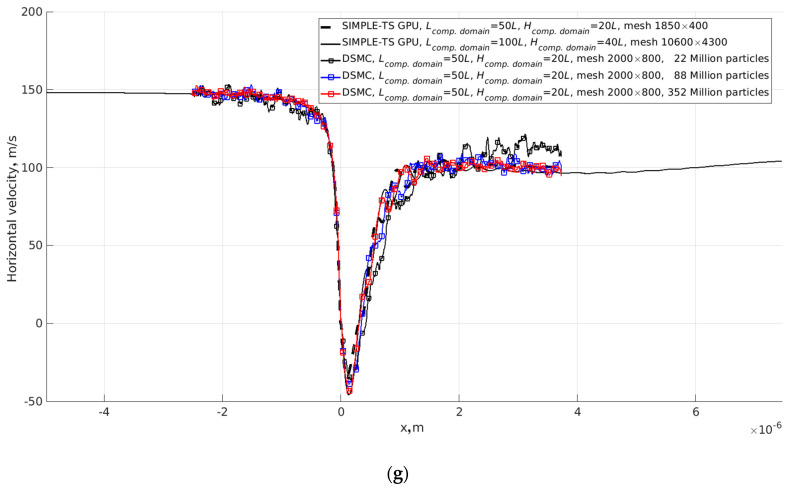
(**a**) Macroscopic field of horizontal velocity; (**b**) SIMPLE-TS staggered grid with 10,600 × 4300 cells; (**c**) zoomed-in SIMPLE-TS staggered grid with 10,600 × 4300 cells around cantilever; (**d**) mesh near cantilever of DSMC; (**e**) zoomed-in mesh near cantilever of DSMC around cantilever; (**f**) application of TAS in basic cells; and (**g**) horizontal velocity profiles at center of flat plate along *x*-axis. Profiles were obtained by SIMPLE-TS GPU (continuum model) and DSMC.

Selecting appropriate flow parameters is essential to the investigation. Figure 1 shows the geometry of a rarefied gas flow past an elastic cantilever beam normal to the free stream. In the considered test case, an elastic beam is replaced with a rigid flat plate. The computational requirements of DSMC increase when the Knudsen number decreases because it approaches the continuum regime, and DSMC has to increase the number of particles in the simulation. Taking into account flow regimes [29,30] and the investigation of flow regimes in microfluidics presented in [31], we selected gas properties to obtain a stable Karman vortex street behind the plate (see Figure 2a) and complete the calculations within a reasonable time. Figure 2a shows the field of horizontal velocity of fully established unsteady flow.

The parameters of the considered problems are as follows: Knudsen number is 0.014, inlet velocity is 0.4 times Mach (148.4 m/s), reference temperature ($T_{reference}$) is 288 K, reference pressure ($p_{reference}$) is 53.4 kPa, the length of the flat plate (*L*) is 8.89 µm, the thickness of the plate (*h*) is 0.02 *L*, and the temperature of the flat plate is constant and equal to the reference temperature. Knudsen, Mach, and Reynolds numbers are related as follows (see [32]):

$$Re=\frac{16}{5\pi }\sqrt{\frac{\gamma^{g}\pi }{2}}\frac{Ma}{Kn}\approx \sqrt{\frac{\gamma^{g}\pi }{2}}\frac{Ma}{Kn}$$

The Reynolds number for the considered case is 47.

As the Knudsen number is sufficiently small, we used the continuum model to validate the macroscopic fields obtained by DSMC code and determine a sufficient size for the computational domain. As we pointed out in the Introduction, based on the results in [23], the macroscopic fields of DSMC and Navier–Stokes equations for the considered Knudsen number were sufficiently close. The continuum model was based on Navier–Stokes–Fourier equations (see [33]) and calculated with the graphics processing unit (GPU) version of the finite volume method SIMPLE-TS (see [34,35]).

The initial boundary conditions for SIMPLE-TS and DSMC are the same: the velocities are equal to the inlet velocities, pressure is equal to the reference pressure, and temperature is equal to the reference temperature. When the simulation starts, there is a transition period before the established nonstationary regime is reached.

All considerations were taken after the transition period, that is, around 10 µs. The analyses were obtained for the period of 14.8 µs of the established unsteady regimes. The beginning of the time axis of the results was set at the beginning of the established regime, i.e., at the end of the transition regime for each case.

Figure 2g shows a comparison between the results obtained by DSMC and SIMPLE-TS. We calculated two cases using the continuum model to study mesh convergence and determine the sufficient size of the computational domain. The computational domain of the first case consisted of length *L_comp. doman_* = 50 *L* and height *H_comp. domain_* = 20 *L*. We used a Cartesian staggered mesh (see Figure 2b,c). The cantilever was colored blue. The minimal and maximal steps along the *x*-axis were $\Delta x_{\min}^{g}=0.005\;L$ and $\Delta x_{\max}^{g}=0.05\;L$, respectively, and the step along the *y*-axis was uniform $\Delta y^{g}=0.05\;L$. The obtained mesh had 1850 × 400 cells. The time step was $\Delta t^{SIMPLE-TS}=2.2\times 10^{-11}\;s$. The computational domain of the second case with finer mesh had length *L_comp. doman_* = 100 *L* and height *H_comp. domain_* = 40 *L*. We used a Cartesian staggered mesh with minimal and maximal steps along the *x*-axis, $\Delta x_{\min}^{g}=0.005\;L$ and $\Delta x_{\max}^{g}=0.01\;L$, respectively; minimal and maximal steps along the *y*-axis $\Delta y_{\min}^{g}=0.005\;L$ and $\Delta y_{\max}^{g}=0.01\;L$, respectively, and the mesh had 10,600 × 4300 cells. The time step was equal to the time step of the first case, $\Delta t^{SIMPLE-TS}=2.2\times 10^{-11}\;s$. The finer mesh in both cases was used around the flat plate. Note that the second case used a finer mesh and larger computational domain. There was excellent agreement between the two continuum cases. Therefore, the computational domain and accuracy of the mesh of the first case were sufficient to obtain the correct results of the considered problem.

DSMC used a hard sphere model of a monoatomic gas and the SBT collision scheme [36]. We modelled air with average molar mass 29 gm/mol, reference viscosity $18.2\times 10^{-6}\;\mathrm{Pa}\;s$, and corresponding particle diameter $3.7\times 10^{-10}\;m$. The diffuse reflection boundary condition was used at the plate walls. DSMC used an adaptive mesh (see Figure 2d–f). The mesh was separated into two types. We used adaptive unstructured mesh in a small domain near the flat plate (colored blue in Figure 2d,e). The adaptive unstructured mesh was generated using the Delaunay algorithm by the Gmsh mesh generator [37] with C++ API functions implemented in our high-performance computing parallel C++ DSMC code. The mesh in the other part of the computational domain was a Cartesian uniform mesh with Transient Adaptive Sub-cell (TAS) [38]. We called the cells of Cartesian uniform mesh “basic” cells. The idea of TAS is to separate each basic cell of the Cartesian uniform mesh into subcells dynamically (see Figure 2f). We used uniform mesh with an equal number of cells in each direction, called *level*. The *level* at each basic cell was calculated as follows:

$$level=\left\lfloor \sqrt{PPC_{}^{\left(basic\right)}/PPC_{\min}^{}}\right\rfloor$$

where level is the TAS level of divisions, representing an integer that denotes the number of subcells in each direction of the basic cell, i.e., for the 2D case, the local mesh has level×level cells, $PPC_{}^{\left(basic\right)}$ is the number of particles per basic cell, and $PPC_{\min}^{}$ is the minimal average number of particles per subcell. This criterion keeps the number of particles per cell as close to constant as possible. The collision procedure is executed in each subcell considering particles in it. We followed the recommendations of the correctness of the SBT collision scheme presented in [39], and we kept an adaptive mesh with an average of three particles per cell ($PPC_{\min}^{}=3$).

Free-stream boundary conditions are an important part of the DSMC simulation. We used boundary cells of the computational domain as reservoirs to generate particles with properties partly determined by the solution. At the inlet at every time step, we generated a new set of particles for each basic cell with Maxwellian velocity distribution and fixed the gas properties as: $u_{inlet}^{g}=0.4\;Mach=148.4\;\;m/s$, $v_{inlet}^{g}=0$, $w_{inlet}^{g}=0$, $p_{inlet}^{g}=p_{reference}^{g}$, and $T_{inlet}^{g}=T_{reference}^{g}$, where $u_{inlet}^{g}$, $v_{inlet}^{g}$, and $w_{inlet}^{g}$ are velocities along the *x*-, *y*-, and *z*-axis, respectively; $p_{inlet}^{g}$ is the pressure at the inlet; $T_{inlet}^{g}$ is the temperature at the inlet. Note that in molecular simulations, each particle has three velocity components independently of the considered macroscopic problem. At the outlet, we applied the following boundary conditions: $\partial u^{g}/\partial x=0$, $\partial v^{g}/\partial x=0$, $\partial w^{g}/\partial x=0$, $p_{outlet}^{g}=p_{reference}^{g}$, and $T_{outlet}^{g}=T_{reference}^{g}$. The top boundary conditions are: $u_{top}^{g}=0.4Mach$, $\partial v^{g}/\partial x=0$, $\partial w^{g}/\partial x=0$, $p_{top}^{g}=p_{reference}^{g}$, and $T_{top}^{g}=T_{reference}^{g}$. The bottom boundary conditions correspond to the top boundary conditions. Detailed information about the boundary conditions can be found in [40].

The size of the DSMC computational domain was length *L_comp. doman_* = 50 *L* and height *H_comp. domain_* = 20 *L*, the same as the size in the first continuum case. We selected this size because the results showed it was sufficient to neglect the influence of the computational domain boundaries. We used basic mesh with 2000 × 800 cells and obtained results with 22, 88, and 352 million particles and time steps of $\Delta t^{DSMC}=4\times 10^{-11}\;s$, $2\times 10^{-11}\;s$, and $10^{-11}\;s$, respectively. As the number of particles increased, the number of adaptive cells increased, and the time step decreased. The time step of each considered case was sufficiently small to satisfy the Courant–Friedrichs–Lewi condition, i.e., none of the particles in the computational domain flew over a cell without executing the collision procedure. We used one trajectory and time-averaging of 1000 time steps to obtain macroscopic properties. The considered number was sufficiently small to keep changes in macroscopic fields such as Karman vortex street noticeable and sufficiently large to obtain smoother macroscopic fields.

Figure 2g shows comparisons of SIMPLE-TS GPU (continuum model) and DSMC. The two profiles obtained by SIMPLE-TS GPU are very close to each other. Therefore, according to this comparison, the spatial accuracy and the size of the computation domain used by the first continuum case obtained correct results. We investigated DSMC convergence by comparing the results obtained with 22, 88, and 352 million particles. Note that we keep the number of basic cells constant, but the TAS level (the number of subcells per basic cell) was increased proportional to the number of particles. DSMC velocity profiles converged to continuum model profiles. The compared profiles show that the DSMC simulation with 88 million particles obtained sufficiently accurate results. Nevertheless, as our investigation was intended to study the influence of VIV, we paid particular attention to the comparison of frequencies and amplitudes of VIV.

**Figure 3:**
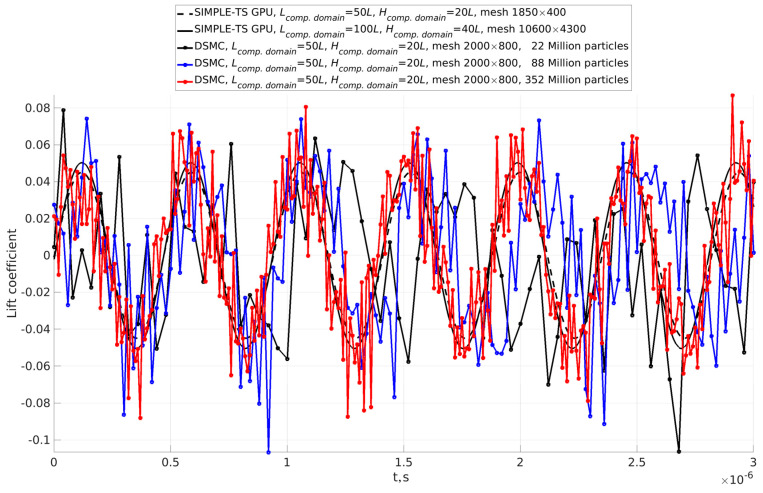
Comparison of convergence of time series of lift coefficients at established regime obtained by continuum approach (SIMPLE-TS) and DSMC.

We analyzed the influence of the Karman vortex street obtained by the continuum solver and DSMC. To that aim, we used time series of lift coefficient (along the *y*-axis, $C_{L}^{g}$) (see Figure 3). $C_{L}^{g}$ was selected for analysis instead of drag coefficient (the coefficient along *x*-axis) because of the lower noise. $C_{L}^{g}$ was defined as follows:

(1) $$C_{L}^{g}=\frac{F_{L}^{g}}{\frac{1}{2}\rho_{reference}^{g}{\left(u_{inlet}^{g}\right)}^{2}S^{g}},$$

where $F_{L}^{g}$ is the lift force; $\rho_{reference}^{g}$ is the reference density obtained using the equation of an ideal gas: $\rho_{reference}^{g}=p_{reference}^{g}/(R^{g}\;T_{reference}^{g})$; $R^{g}$ is the gas constant; $S^{g}$ is the projected area. The Strouhal number is a dimensionless number describing oscillating flows, and it was defined as:

(2) $$St^{g}=\frac{f_{C_{L}}^{g}L}{u_{inlet}^{g}}$$

where $f_{C_{L}}^{g}$ is the frequency of the lift coefficient.

**Figure 4:**
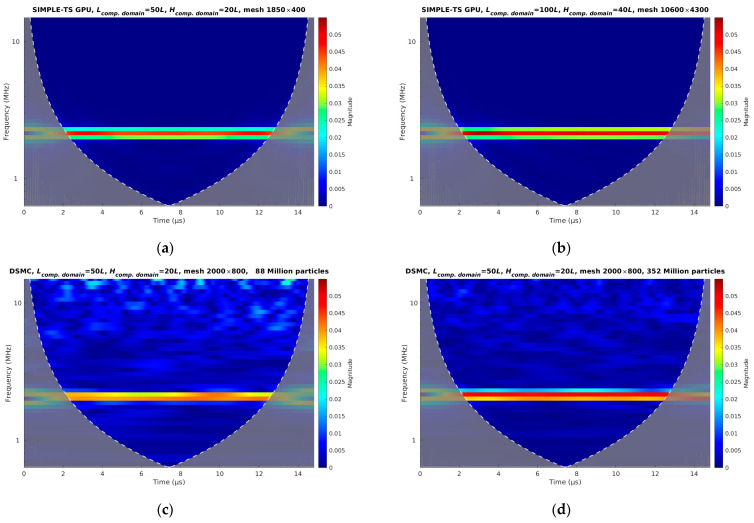
Comparison of continuous 1D wavelet transform with bump wavelet to analyze time series of lift coefficients at established regime obtained by continuum approach (SIMPLE-TS) on computational domain with size (**a**) 50 *L* × 20 *L* and mesh with 1850 × 400 cells and (**b**) 100 *L* × 40 *L* and mesh with 10,600 × 4300 cells, and DSMC obtained on computational domain with size 50 *L* × 20 *L* and (**c**) 88 million particles and (**d**) 352 million particles.

We analyzed $C_{L}^{g}$ time series with fast Fourier transform (FFT) and continuous 1D wavelet transform (CWT) with the bump wavelet. It is known that FFT gives good results about frequencies, while the amplitude is determined quantitatively. We analyzed data with CWT to overcome the disadvantages of FFT (see Figure 4 and Table 1). Figure 4 shows the CWT of the time series of the lift coefficient obtained by the continuum model and DSMC. A notable difference between DSMC with 88 and 352 million particles can be seen. The results of all calculated cases are presented in Table 1, which shows a comparison of frequencies and amplitudes obtained by FFT and CWT. The amplitudes of $C_{L}^{g}$ time series shown in Figure 3 are significantly different from those obtained by FFT, as expected. On the other hand, the amplitudes obtained by CWT are in excellent agreement with the $C_{L}^{g}$ time series.

Based on the obtained frequencies, we calculated the Strouhal numbers of the considered cases. The results obtained by SIMPLE-TS GPU are very close to each other, confirming that the computational domain was of sufficient size. Frequencies and amplitudes obtained by DSMC cases were not as close as horizontal velocity profiles but converged to results obtained by a continuum model. The frequency and amplitude obtained by DSMC with 352 million particles and the second case of the continuum model are in excellent agreement; the difference is less than 0.5% of the frequencies and 2% of the amplitudes. We decided to use DSMC simulations with 352 million particles because in our study the accuracy of VIV is important. According to the results, we believe that our results are accurate within a couple of percent (2–3%).

### 2.2. Mathematical Model of Elastic Obstacle

The beam considered in this work had the following material and geometrical properties: Young’s modulus E = 5.5 × 10^11^ N/m^2^, density *ρ* = 1.58 × 10^4^ kg/m^3^, Poisson ratio ν = 0.2445, beam length *L* = 8.89 × 10^−6^ m, thickness *h = L*/50, and beam width *b* = 2 × *h*. The mechanical properties of the beam material correspond to tungsten carbide.

Considering the dimensions of the elastic element obstructing the fluid flow, it was obvious that the most adequate theoretical model to use would be beam theory.

The beam was thin enough, but to determine which beam theory to use, we checked the applicability of the classical Euler–Bernoulli beam theory and the first-order shear deformation theory of Timoshenko for the selected geometry of the beam and material properties. The main dynamic characteristics of a structure are the natural frequencies of vibration. For a cantilever beam described by Euler–Bernoulli beam theory, there are explicit formulas for computing the natural frequencies in Hz:

(3) $$\omega_{1}=\frac{3.516}{2\pi }\sqrt{\frac{EI}{mL^{4}}},\;\omega_{2}=\frac{22.0354}{2\pi }\sqrt{\frac{EI}{mL^{4}}},\;\omega_{3}=\frac{61.6972}{2\pi }\sqrt{\frac{EI}{mL^{4}}},\;m=\rho A,\;A=bh$$

where

(4) $$I=\frac{bh^{3}}{12}$$

They are solutions of the following frequencies equation:

(5) cosβLcoshβL=−1

where

(6) $$\beta_{n}^{4}=\frac{\omega_{n}^{2}\rho A}{EI}$$

In order to choose the proper theory, the first three natural frequencies were computed according to the beam theories of Euler–Bernoulli and Timoshenko. The same frequencies were computed using a commercial finite element program, MSC Nastran.

It turns out that well-known programs such as MSC Nastran and ANSYS cannot work with objects with such small dimensions. That is why the comparison of frequencies was performed on a beam with geometrical dimensions 10^5^ times bigger than the real ones.

A finite element model of a beam was created by MSC Patran using 41 linear beam elements and the first 10 natural frequencies were obtained.

The results for the first three natural frequencies obtained in three ways are shown in Table 2.

Here, the frequencies according to Timoshenko beam theory were obtained as a roots of a transcendental equation presented in [41].

It is clearly seen that the first natural frequencies obtained by the Euler–Bernoulli and Timoshenko beam theories for the selected geometrical and physical parameters of the beam are practically identical. Both are also very close to the frequencies obtained by MSC Nastran. This convinced us to use the simplest Euler–Bernoulli beam theory in our computation.

For the real geometry, the beam frequencies are: ω_1_ = 0.13478 × 10^8^ rad/s = 2.1451 MHz, ω_2_ = 0.84467 × 10^8^ rad/s = 13.443 MHz, and ω_3_ = 0.23651 × 10^9^ rad/s = 37.642 MHz.

In [27], the interaction of a cantilever with flow in a micro-channel was analyzed using the geometrically nonlinear version of Euler–Bernoulli beam theory. Taking into account that the load of the flow leads to vibration with small deflections (in comparison with the beam thickness), we decided to use linear beam theory in this work. We believed that this approach would give enough knowledge for the effect we decided to investigate in this study. Moreover, the fact that there are explicit formulas for the natural frequencies of a Euler–Bernoulli cantilever is a big advantage for our study, as can be seen below.

The governing equations of beam motion in terms of displacement are well known, and can be written in the form [42]:

(7) $$\frac{\partial^{4}w}{\partial y^{4}}+c_{v}\frac{\partial w}{\partial t}+\frac{\rho A}{EI}\frac{\partial^{2}w}{\partial t^{2}}=\frac{p(y,t)}{EI}$$

In Equation (1), *w* is the transverse beam displacement (along the *x*-axis), *ρ* is the density of the beam material, *p*(*y*,*t*) is the transverse loading of the beam, *A = b × h* is the area of the beam cross-section, *t* is the time, and *c*_v_ is the material damping coefficient.

The boundary conditions for a cantilever (clamped at one end and free at the tip) can also be found in Meirovitch’s books [42]:

(8) $$\begin{array}{l} w(0,t)=0,\;\frac{\partial w(0,t)}{\partial y}=0\\ \frac{\partial^{2}w(0,t)}{\partial y^{2}}=0,\;\frac{\partial^{3}w(0,t)}{\partial y^{3}}=0 \end{array}$$

For the solution of the partial differential Equation (7), a reduced model of the beam was created [27]. The solution is sought by the series

(9) $$w(y,t)=\sum_{n=1}^{N_{f}}w_{n}(y)q_{n}(t).$$

where *w*_*n*_(*y*) denotes space functions representing vibration modes that should satisfy the geometrical boundary conditions, *q*_*n*_(*t*) denotes time functions, and $N_{f}$ is the number of modes in the expansion. The natural modes of vibrations *w_n_* have the expression:

(10) $$w_{n}(y)=A_{n}\left[\sin\beta_{n}y-\sinh\beta_{n}y-B_{n}(\cos\beta_{n}y+\cosh\beta_{n}y)\right]$$

where

$$B_{n}=\frac{\sin\omega_{n}L+\sinh\omega_{n}L}{\cos\omega_{n}L+\cosh\omega_{n}L}$$

Following the procedure presented in [27,41,42], the following system of ordinary differential equations was obtained:

(11) $${\ddot{q}}_{n}(t)+2\xi_{n}\omega_{n}{\dot{q}}_{n}+\omega_{n}^{2}q_{n}(t)=F_{n}(t),$$

Here, $\xi_{n}$ represents modal damping parameters and

(12) $$F_{n}(t)=\int_{0}^{L}w_{n}^{}(y)\;\;[P(y,t)]\;dy,\;\;P(y,t)=\frac{p(y,t)}{EI}$$

Here, similar to [1], three modes are used in the expansion (9), i.e., *N_f_* = 3.

Equation (11) is a linear system of ordinary differential equations and its solution can be obtained analytically. In the present work, the system of ordinary differential equations (Equation (11)) was solved numerically by the Gears method [43]. The integrals in Equation (12) are solved numerically using Simpson’s rule.

### 2.3. Verification of the Beam Model

The reduced model was verified by comparing the results from the forced vibration of the beam obtained by the developed model and those obtained by numerical integration of the full model with MSC Nastran. It turned out that the well-known general-purpose programs MSC Nastran and ANSYS cannot work with structures that have dimensions, which we have intended to analyze in our study. It was not possible to create a model of a beam with length *L* = 8.89 × 10^−6^ m. That is why we decided to verify our theoretical model using a beam with 10^5^ times bigger dimensions, keeping the relations *h/L* = 0.02 and *b/h* = 2. For the linear case, the solutions of the vibration problems for beams with proportional dimensions keep the same proportions. It is not possible (or it is very difficult) to simulate in a commercial finite element method (FEM) program the dynamic load that arises in the process of the rarefied gas flow interacting with the beam. That is why just two kinds of dynamic loading, step loading and harmonic loading, were considered. These regimes of loading are somewhat similar to gas loading, because they can start suddenly (step loading) and can have some periodicity because of the vortex (harmonic loading).

In the case of step loading, the beam was loaded with

(13) $$p(y,t)=\left\{\begin{array}{c} \;0\;\;\mathrm{for}\;t\leq 0\\ p_{0}\;\mathrm{for}\;t>0 \end{array}\right.$$

while for the second case the loading was described by the formula:

(14) $$p(y,t)=p_{h}\sin(\omega_{e}t)$$

In both cases, the loading was uniformly distributed along the beam length.

In (14), *p_h_* is the amplitude of the harmonic loading and *ω_e_* is the excitation frequency.

**Figure 5:**
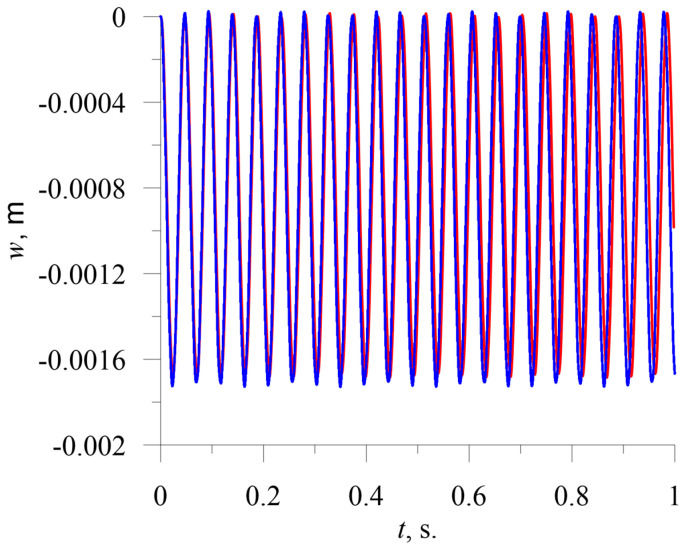
Time–history diagram of response of tip of beam subjected to uniformly distributed step load with *p*_0_ = 100 N/m. Blue indicates presented model, red indicates MSC Nastran.

The results obtained for the forced vibration of the beam subjected to uniformly distributed step loading are shown in Figure 5.

**Figure 6:**
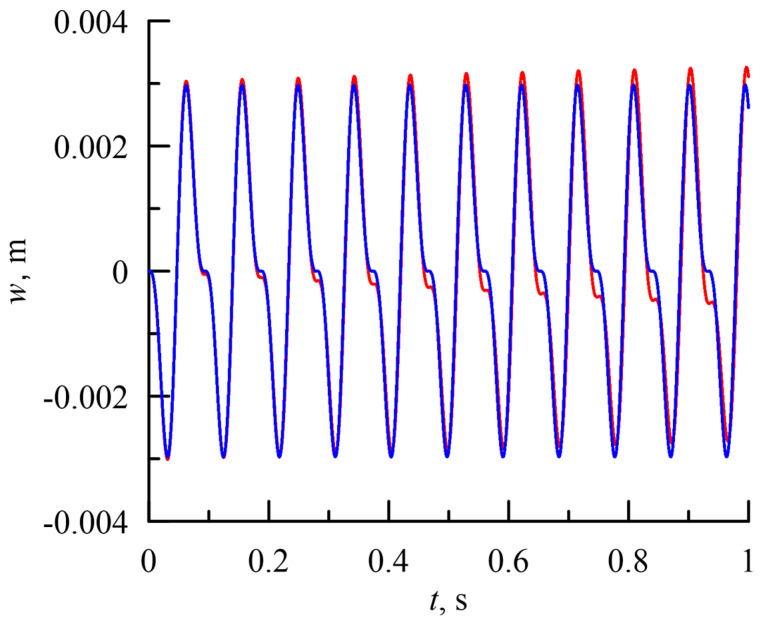
Time–history diagram of response of tip of beam subjected to uniformly distributed step load with *p*_*h*_ = 200 N/m and ω_*e*_ = 67.4 rad/s.

It is clearly seen that the results of the computations using the current model and the MSC Nastran model are almost identical. Similarly, good agreement can be observed in the case of harmonic loading (Figure 6). In this case, the differences in the results between the models are a little bigger, but still very small. The excitation frequency was chosen to be half of the second natural frequency (or between the first and second natural frequency). In this case, a larger amplitude of loading was chosen, *p*_*h*_ = 200 N/m. Both cases confirm the applicability of the reduced model of beam vibration.

It should be noted that the selected loading in this case led to vibrations with comparatively small amplitudes with respect to the thickness, and the influence of the nonlinear terms was small. The real case studied in this work corresponds to the examples. Regardless of the small dimensions of the beam, the forces exerted by the rarefied gas flow led to oscillations with small amplitudes with respect to *h*. This allows us to conclude that for the selected problem it is enough to consider only the linear case (in contrast to the case we considered in [27]) and not take into account large deflections. Using this linear model, Equation (11) is much simpler than the equation solved in [27], which included 10 cubic terms.

## 3. Results and Discussion

The aim of our research was to study, within the framework of FSI, the amplitude of displacement of an elastic beam when the frequency of VIV from a Karman vortex street is close or equal to the first natural frequency of the beam. Such investigation is complicated because we have to analyze the governing parameters of two interacting media: gas flow and elastic cantilever beam. Dimensional analysis of the gas flow and analytical expression of the first natural frequency of the beam are important in determining the independent governing parameters of the coupled system.

### 3.1. Dimensional Analysis

Dimensional analysis is a powerful tool for evaluating a problem and clarifying the number of governing parameters and their role. Dimensional analysis helped us to determine the governing parameters and their possible variations. First, we analyzed the fluid part of the problem. We considered similar geometric systems and monatomic ideal gas of hard spheres. The time evolution of the continuum model used in DSMC validation was described based on the Navier–Stokes–Fourier equations for a compressible viscous gas, with transport coefficients determined by the first approximation of Chapman–Enskog theory for low Knudsen numbers [33]. The Chapman–Enskog theory provides a framework in which equations of hydrodynamics for a gas can be derived from the Boltzmann equation. On the other hand, the DSMC method uses probabilistic Monte Carlo simulation to solve the Boltzmann equation for finite Knudsen number fluid flows [28]. As the considered Knudsen number was sufficiently small, we performed a dimensional analysis of a continuum model.

The fluid model is unsteady, viscous, compressible, and heat conducting, does not consider an external field, and has a constant temperature on the wall surfaces and a constant specific heat ratio. We followed the considerations described in [44,45]. In general, such a system depends on Mach number, Reynolds number, Strouhal number, Froude number, specific heat ratio, Prandtl number, and the ratio between the temperature at infinity and wall temperature. We reduced the number of governing parameters that influence the solution by considering the specifics of the investigated problem. The Froude number was not considered because we did not consider the external field. We fixed the fluid to a monatomic ideal gas of hard spheres, with specific heat ratio (*γ^g^*) equal to 5/3, and fixed specific heat and viscosity at infinity; therefore, the Prandtl number was also constant. The temperature of the walls and at infinity was fixed at 288 K; therefore, the temperature ratio was also constant. Finally, only Mach, Reynolds, and Strouhal numbers were left. As we fixed the geometry, the Strouhal number depended on the Mach and Reynolds numbers and nothing else. Finally, the governing parameters of a considered gas as part of the coupled system were Mach and Reynolds numbers.

As we were considering micro-gas flow, it was more convenient to use the Knudsen number instead of the Reynolds number. Knudsen, Mach, and Reynolds numbers are related, as was pointed out.

Finally, the considered system depended on the Mach and Knudsen numbers, and the obtained Strouhal number was a function of them because the influence of beam displacement on the macroscopic fields was negligible and could be neglected for our analysis.

Our aim was to investigate the case where the flow frequency is close or equal to the first natural frequency of the beam. The possible parameters that could be varied were determined by equating the frequency of $C_{L}^{g}$, i.e., the frequency of VIV ($f_{C_{L}}^{g}=St^{g}\;u_{inlet}^{g}/L$ from Equation (2)) and the analytical expression of the first natural frequency of the elastic beam:

(15) $$\frac{St^{g}\;u_{inlet}^{g}}{L}=\frac{3.5160}{2\pi }\sqrt{\frac{E\;I}{\rho hb\;L^{4}}}=\frac{1.01498\;\;c_{h}}{2\pi L}\sqrt{\frac{E}{\rho }}$$

In these formulas, Equation (16) and the expression *c*_*h*_ = *h/L* are used.

After simplification, we have the following equation:

(16) $$St^{g}\;u_{inlet}^{g}=\frac{1.01498}{2\pi }\;\;c_{h}\sqrt{\frac{E}{\rho }}$$

The obtained simplified Equation (16) consists of variables that can be modified to study the resonance of the gas–elastic beam system. We supposed that resonance should appear when Equation (16) is satisfied within the range $\omega_{1}/f_{C_{L}}^{g}\in (0.75,1.25)$.

Note that the length of the beam is not included independently of the Knudsen number, so it is not possible to change the length and influence the $C_{L}^{g}$ frequency without changing the Knudsen number. The left-hand side consists of variables related to the gas, while the right-hand side consists of variables related to the elastic beam. For simplicity, we chose to vary one parameter and discuss the possibilities here. The Strouhal number depends on the Mach and Knudsen numbers, and we can vary them to change the VIV frequency to study the resonance of an elastic beam. Such variation is complicated, because the frequency of the vortex shading depends on the Mach and Knudsen numbers in a nonlinear way. Furthermore, varying these parameters brings additional difficulties. If we vary the Knudsen number, we will increase the computational requirements significantly when that number decreases. If we vary the Mach number, we have to consider that local shock waves for the considered geometry arise at around Mach 0.6 to 0.7. The fluid flow will change from subsonic to transonic. Reducing the Mach number too much will increase the computational requirement of DSMC because of the noise at low speeds with this method. Therefore, we could vary the Mach number in a very narrow range. The parameters of an elastic beam are thickness, *E*, and ρ. We decided to fix the material properties of the modeled elastic beam to the properties of real material. The only parameter left was *c_h_*. Finally, we decided to fix gas velocity (Mach number), rarefaction (Knudsen number), and elastic beam material, and to vary elastic beam thickness *h* (or *c_h_*).

### 3.2. Results

We investigated VIV in an elastic beam when the frequency of the vortex is close or equal to the first natural frequency of the beam. According to the dimensional analysis, we decided to fix the Mach and Knudsen numbers and the material of the elastic beam and to vary the thickness of the elastic beam. The considered problem setup was the same as the validation of DSMC with 352 million particles, with time step $\Delta t^{DSMC}=10^{-11}\;s$, and we kept an adaptive mesh with an average of three particles per cell ($PPC_{\min}^{}=3$). The temperature of the elastic beam was constant and was equal to the reference temperature. The diffuse reflection boundary condition was used at the cantilever walls. Here, instead of a rigid plate, we used an elastic cantilever beam with fully coupled gas–elastic beam interaction. We used one trajectory and time averaging of *N^DSMC^* = 20 time steps. *N^DSMC^* was selected small to account for non-equilibrium effects on the surface of the beam. At every *N^DSMC^* time step calculated by DSMC, the motion of the elastic cantilever beam was modelled using $\Delta t_{}^{C}=4\times 10^{-11}\;s$ time step for *N^C^* = 5 time steps. Detailed information about the numerical approach is presented in [27].

As a material, we selected tungsten carbide (WC) with *E*_WC_ = 550 GPa and $\rho_{WC}=15.8\;g/{\mathrm{cm}}^{3}$. The damping coefficient was set to *ξ* = 0.001. Taking into account the obtained frequency of $C_{L}^{g}$, we could calculate the elastic beam thickness (*c_h_*) from Equation (16) and observe that the first natural frequency was equal to the frequency of VIV of $C_{L}^{g}$ when *c_h_* = 0.0201.

The results were obtained after the transition period, which was around 12 µs. The analysis was performed for the 10 µs period of established unsteady regime. The beginning of the time axis of the results was set at the beginning of the established regime, i.e., at the end of the transition regime for each case.

**Figure 7:**
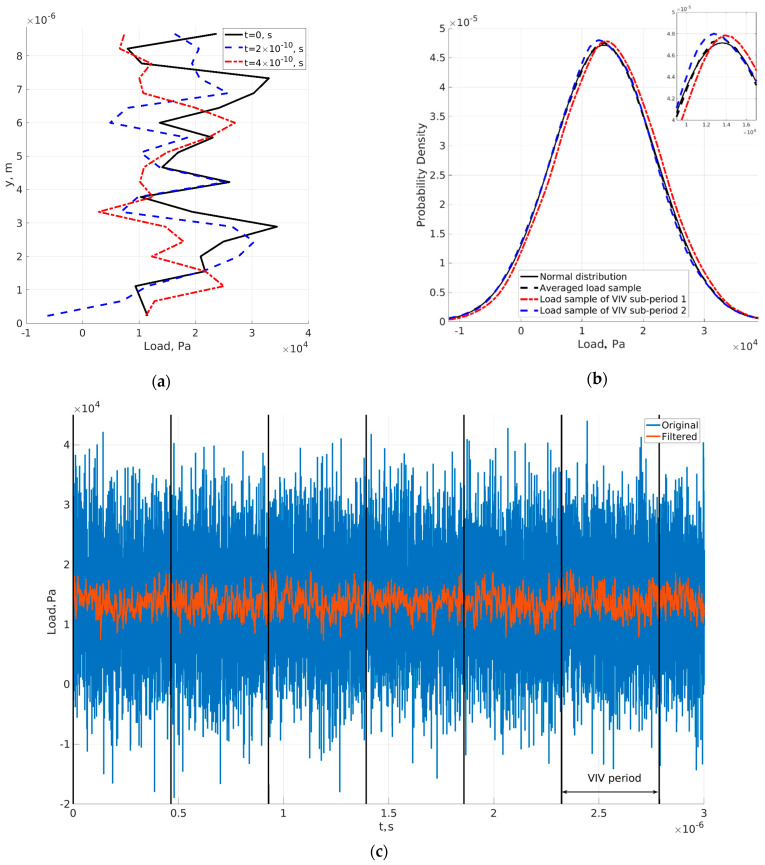
Load on beam. (**a**) Load along beam length for three sequential time steps; (**b**) probability density function of load of a node near the tip; (**c**) load at a node near the tip in time. Beam thickness is *c_h_* = 0.0201.

Figure 7 shows the load of the fluid flow. Figure 7a shows the load along the beam length for three sequential time steps and Figure 7c shows the load at the tip of the beam in time. The load was filtered by a median filter, and the periods of VIV can be seen. As we can see, the load is very irregular and looks stochastic.

Figure 7b shows the load distribution at one node of the beam, where the plotted normal distribution is based on a load sample and a kernel smoother was used for the other plots in order to make the data comparison easier. When the load sample represented data from all considered periods, the influence of vortex shedding vanished, and the load distribution was very close to normal. We separated the load into two periods according to the tip movement. The first period was when the tip was moving toward the positive direction of the *x*-axis, and the second was in the opposite direction. The distributions of the two loads deviated from normal distribution. The reason for the difference was the influence of vortex shedding, i.e., the periodic detachment of vortices behind the tip of the cantilever. Continuum models cannot obtain the statistical distribution of the load. They obtain the oscillation of vortex shedding based on the mean values of the presented distributions. In microfluidics, non-equilibrium surface effects play an important role and can be captured by DSMC, as mentioned earlier. DSMC (molecular approach) can obtain statistical load distribution, and the results are valid for all regimes.

On the other hand, the computational requirements of DSMC and continuum solver (SIMPLE-TS GPU) depend on the required accuracy and the problem to be calculated. For the calculated problem with used accuracy, the calculation times of both solvers are close. A detailed comparison is outside the scope of this paper.

It is interesting to see the response of the beam to such a complicated load.

**Figure 8:**
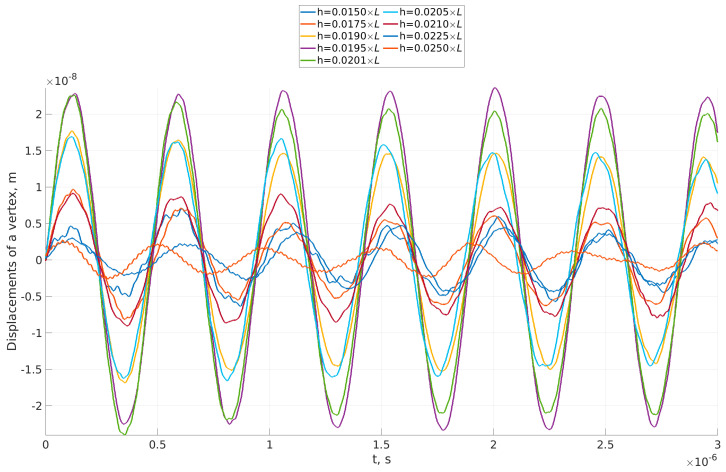
Time–history diagram of deflection of tip of beam for beams with 9 thicknesses.

The results, showing the response of the beam with different thicknesses, are presented in Figure 8. As expected, the deflection of the beam depends on the thickness. The beam vibrates with almost periodic vibration. The vibrations with increased amplitude are smooth, while some perturbation in each period is observed in those with smaller amplitude. This shows that when the flow induces vibrations with larger amplitudes (depending on the thickness of the beam), the irregular character of the loading does not influence the periodic vibration of the beam. Another, more interesting fact is that increasing the thickness of the beam does not in all cases decrease beam deflection. The thickness of the beam was varied, and nine values were used to calculate the steady-state response of the beam to VIV.

The amplitudes of the beam can be seen in the time–history diagrams shown in Figure 8. To be more precise, however, a wavelet transform was performed, and the frequencies and amplitudes of the responses were calculated.

**Figure 9:**
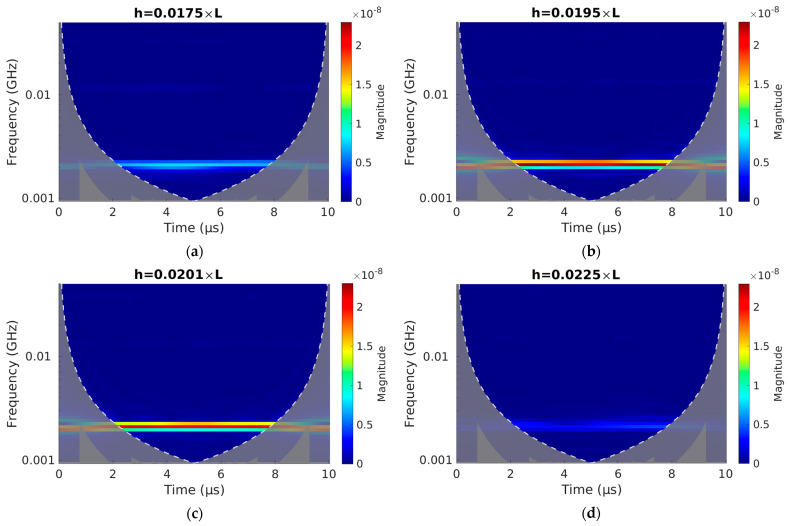
Continuous 1D wavelet transform with bump wavelet analysis of deflection of tip of beam for thickness of (**a**) 0.0175 *L*, (**b**) 0.0195 *L*, (**c**) 0.0201 *L*, and (**d**) 0.0225 *L*.

Figure 9 shows the wavelet transforms of the time series of the response of the tip of the beam with four different beam thicknesses.

We can expect that decreasing the thickness (thus decreasing the beam rigidity) will lead to increased natural frequencies and amplitudes of vibration. Indeed, this is valid until a certain thickness. When the thickness reaches values close to the first natural frequency, the amplitude sharply increases. Further decreasing the beam thickness leads to decreased amplitude of beam vibration. This is, in some sense, counterintuitive behavior.

**Figure 10:**
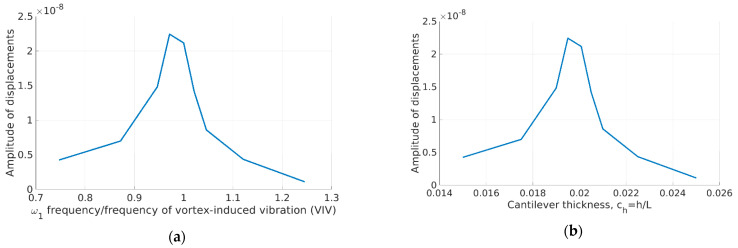
Amplitude of displacement of beam as a function of (**a**) frequency ratio (first natural frequency over VIV frequency) and (**b**) cantilever thickness.

This phenomenon is shown in two equivalent figures, where curves are identical. Figure 10a shows the influence of the first natural frequency of the beam on the maximal amplitude of the vibration, and Figure 10b shows the dependence of the maximal amplitude on the thickness of the beam. As long as the dependence of beam thickness and its first natural frequency is linear, the curves are identical. We have shown both of them in order to be clear that increasing the thickness could lead to increased amplitudes of vibration.

This confirms that the rarefied gas flow can produce a resonance in the beam vibration when the frequency of the flow coincides with or is close to the natural frequency of the beam. This could be useful in certain technological applications and should be taken into account in the design of structures that involve interactions with fluid flow.

## 4. Conclusions and Application

VIV in an elastic micro-beam in a resonance zone was investigated. The considered problem was rarefied gas flow past an elastic micro-beam. We considered a fully coupled FSI approach. DSMC was used to model the rarefied flow, while Euler–Bernoulli beam theory described the elastic beam. To obtain a stable Karman vortex street behind the beam and perform the calculations within a reasonable time, we fixed the Mach number as 0.4 and Knudsen number as 0.014. The corresponding Reynolds number was 47. Dimensional analysis of the fluid problem shows that the governing variables are the Mach and Knudsen numbers; therefore, the frequency of vortex shading in a given case depends on these numbers. Linear beam theory gives analytical equations for the natural frequencies of the beam. We used analytical equations and found that the governing parameters of the considered FSI problem are Mach number, Knudsen number, and beam thickness (beam material is fixed). As the Mach and Knudsen numbers govern the flow in a nonlinear way and bring additional computational difficulties, we varied the beam’s natural frequencies by varying the thickness of the beam. In this way, we fixed the frequency of the VIV of the Karman vortex street and varied the natural frequencies of the beam. We found that when the ratio of the first natural frequency and VIV frequency was within the range of 1 ± 0.25, the beam was in resonance and its amplitude of vibration increased significantly.

The phenomenon described here can be used to produce devices applicable to micro- and nanotechnology. The main advantages of such devices could be their small size and the short transition time. On a microscale, the transition period of fluid flows is in the order of microseconds (see Figure 3, Figure 7 and Figure 8). If such a sensor was designed, it could perform tens of millions of real-time measurements per second. This kind of sensor could be used in a boundary layer to detect dynamically different important flow phenomena, such as separation points where backflows appear, i.e., stalls. Sudden layer separation is one reason for a reduction in lifts or so-called stalls. In everyday use, it would be possible to detect dynamic gas properties in a boundary layer on a flying plane or Formula 1 racing car outside the laboratory without additional equipment.

Another application is to function as an energy-harvesting device. Such a device, using the phenomena VIV provokes, could produce the energy necessary to power small devices such as wireless sensors, wireless sensor networks for the Internet of Things, etc. If the material and geometrical properties of the elastic elements were designed in correspondence with the gas flow parameters, the energy harvest could be maximized.

**Figure 11:**
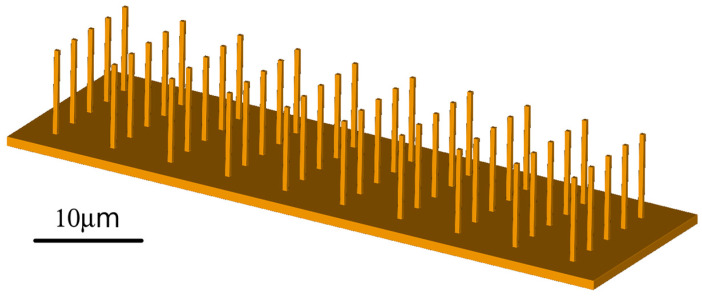
A MEMS contains a cluster of micro-sized elastic beams with different properties and appropriate electronics for wireless data transfer.

Millions of micro-sized elastic beams with different properties could be used to build the sensor. Such MEMS would be similar to the central processing units currently in use. The MEMS could be printed directly on the surface using next-generation 3D printers based on present-day 3D printers for printed electronics and nano-printing together with electronics for wireless data transmission (see Figure 11). Of course, it is also possible to design mixed devices. Some beams could produce energy, while others could collect useful data. These applications are interesting and could be discussed in future works.

## Figures and Tables

**Table 1:** Comparison of frequencies, amplitudes, and Strouhal numbers of lift coefficients at established regime obtained by SIMPLE-TS GPU (continuum approach) and DSMC (molecular approach) and analyzed by FFT and CWT.

Algorithm (Size of Computation Domain, Mesh/Particles)	Fast Fourier Transform			Continuous 1D Wavelet Transform		
	Frequency	Amplitude	*St^g^*	Frequency	Amplitude	*St^g^*
SIMPE-TS GPU (50 *L* × 20 *L*, mesh 1850 × 400 cells)	2.127 MHz	0.04345	0.1274	2.143 MHz	0.0447	0.1283
SIMPE-TS GPU (100 *L* × 40 *L*, mesh 10,600 × 4300 cells)	2.136 MHz	0.04398	0.1279	2.143 MHz	0.0508	0.1283
DSMC (50 *L* × 20 *L*, 22 10^6^ particles)	1.9 MHz	0.01709	0.1138	1.874 MHz	0.03314	0.1122
DSMC (50 *L* × 20 *L*, 88 10^6^ particles)	2.074 MHz	0.03116	0.1242	2.008 MHz	0.04213	0.1202
DSMC (50 *L* × 20 *L*, 352 10^6^ particles)	2.09 MHz	0.04061	0.1252	2.153 MHz	0.04982	0.1289

**Table 2:** Frequencies obtained by Euler–Bernoulli and Timoshenko beam theories and MSC Nastran.

No.	Euler–Bernoulli		Timoshenko		MSC Nastran	
	Rad/s	Hz	Rad/s	Hz	Rad/s	Hz
1	13.478	21.452	13.478	21.445	13.3216	21.3779
2	84.468	134.44	84.466	134.15	84.006	133.7
3	2365.1	376.42	2365.0	374.5	2345.574	373.31

## Data Availability

Not applicable.
